# Targeting RPS6KC1 to overcome enzalutamide resistance in prostate cancer

**DOI:** 10.1186/s40364-025-00822-x

**Published:** 2025-08-23

**Authors:** Fu-Hao Ji, Yu-Hang Qian, Xiu-Chen Guo, Hai-Hong Liao, Jia-Cheng Huang, Zi-Han Xu, Ming-Ming Yu, Yan-Yuan Wu, Jie-Wen Bao, Hao-Jie Chen, Yong-Jiang Yu, Lin Wang

**Affiliations:** 1 Department of Urology, Ren-Ji Hospital, Shanghai Jiao Tong University School of Medicine, Shanghai, 200001 People’s Republic of China; 2 Department of Urology, Shanghai Sixth People’s Hospital Affiliated to Shanghai Jiao Tong University School of Medicine, Shanghai, 200233 People’s Republic of China; 3 Department of Urology, Shanghai 411 Hospital, China Rong Tong Medical Healthcare Group Co. Ltd., Shanghai, 200081 People’s Republic of China; 4 Department of Urology, School of Medicine, Xin-Hua Hospital Affiliated to Shanghai Jiao Tong University School of Medicine, Shanghai, 200092 People’s Republic of China; 5 Department of Urology, School of Medicine, Shanghai Children’s Hospital, Shanghai Jiao Tong University, Shanghai, 200062 People’s Republic of China; 6 Department of Ultrasound in Medicine, Shanghai Sixth People’s Hospital Affiliated to Shanghai Jiao Tong University School of Medicine, Shanghai, 200233 China; 7 Department of Urology, Shanghai Ninth People’s Hospital, Shanghai Jiao Tong University School of Medicine, Shanghai, 200011 People’s Republic of China

**Keywords:** Prostate cancer, Enzalutamide, RPS6KC1, scRNA-seq

## Abstract

The androgen receptor signaling inhibitor enzalutamide (Enz) is one the primary therapeutic drugs for advanced prostate cancer (PCa). Nevertheless, most of patients ultimately develop resistance to Enz. Through an integrated analysis of CRISPR genome-wide and kinome-wide screens, coupled with observations of elevated expression levels in Enz-resistant cell lines and PCa tumor tissues, our study identified *RPS6KC1* as a novel essential gene implicated in Enz resistance. Mechanistically, our research indicates that the Warburg effect induces H3K18 lactylation, which regulates the expression of *RPS6KC1* via the transcription factor P65. Elevated expression of RPS6KC1 was found to recruit PRDX3 to the mitochondria, thereby mitigating ferroptosis. These findings suggest that the H3K18la/NF-κB/RPS6KC1/PRDX3 axis is important for the development of resistance to Enz. Our results suggest that the combination of Enz with targeted RPS6KC1 inhibition or a ferroptosis inducer may represent a promising therapeutic strategy to overcome Enz resistance.

**Graphical Abstract:**

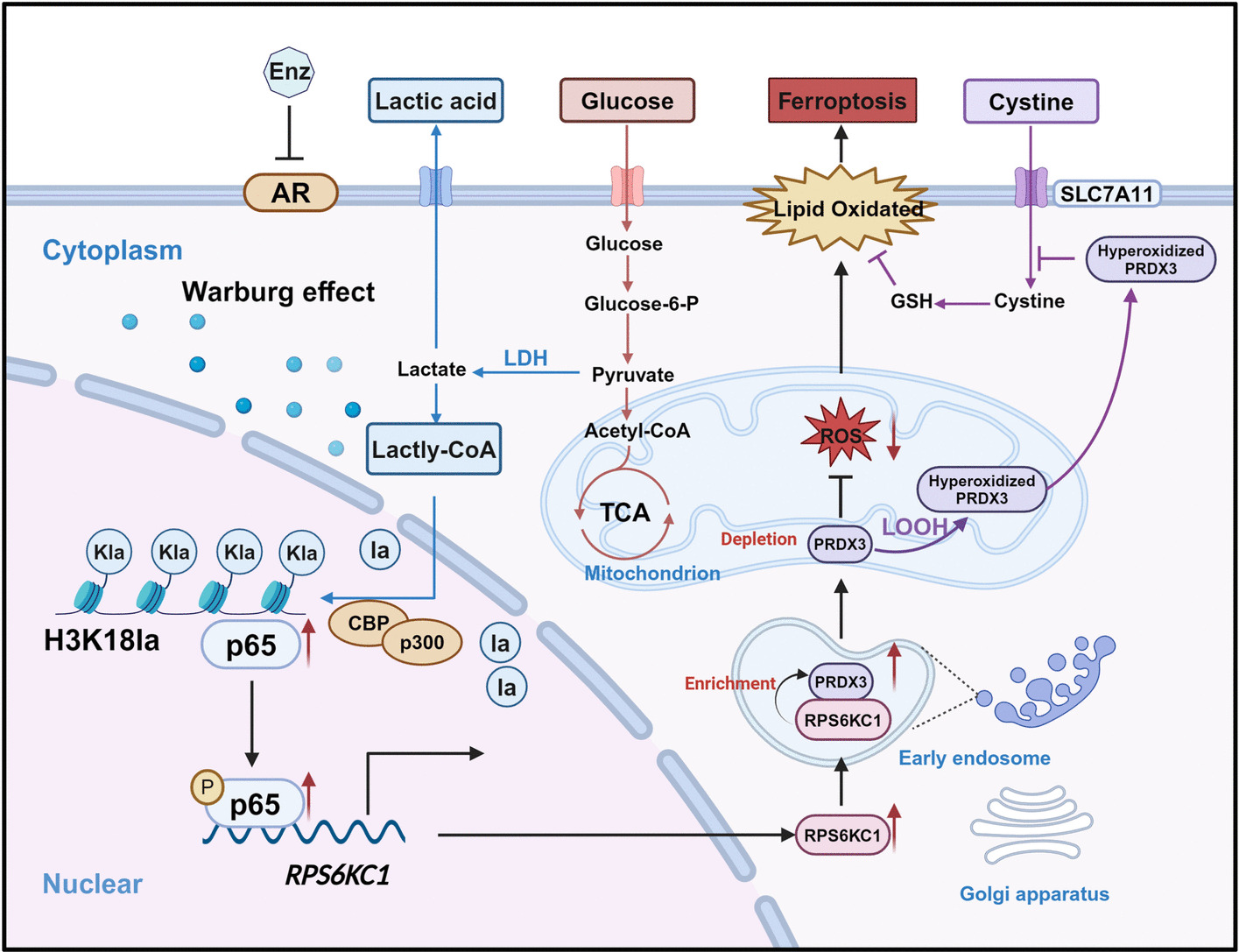

**Supplementary Information:**

The online version contains supplementary material available at 10.1186/s40364-025-00822-x.

## Introduction

Prostate cancer (PCa) is one of the most common cancers in American male [1]. Androgen deprivation therapy (ADT) is the primary treatment for advanced PCa cases, which significantly extends patient’s lifespans [2]. However, the disease often relapses and progresses to castration-resistant prostate cancer (CRPC), underscoring the need for new treatments [3]. Nowadays, powerful second-generation androgen receptor signaling inhibitors (ARSIs), such as Enzalutamide (Enz) and Darolutamide (Daro), have been approved for CRPC and significantly benefits patient prognosis [4]. However, most patients eventually develop resistance to Enz, with few reliable treatment options available, highlighting the need for alternative therapies for Enz-resistant PCa. Studies show that Enz resistance often involves AR-dependent mechanisms like *AR* amplification, *AR* mutations, and *AR* splice variants [5, 6]. Besides, AR-independent mechanisms bypass AR signaling or gain lineage plasticity by expressing neuroendocrine and stem cell genes via epigenetic changes [7–9]. Moreover, recent study suggests that oxidative stress could induces ferroptosis and overcome Enz resistance, though the exact mechanisms are still unclear [10–12].

Protein kinases are crucial for cell regulation, and their mutations can lead to cancer, making them key targets for treatment [13]. RPS6KC1, a protein with a phox homology domain and two pseudo-kinase domains, can bind to sphingosine kinase-1 and co-localize with it on early endosomes [14]. Previous studies indicate that RPS6KC1 binds with SPHK1, influencing key cellular processes like growth, survival, motility, and cytoskeletal changes [15]. Additionally, RPS6KC1 can interact with PRDX3, an antioxidant protein involved in preventing ferroptosis, via its pseudo-kinase domains, and with early endosomes through its PX domain [16]. Although RPS6KC1 has been linked to various solid tumors, its role in ferroptosis and Enz resistance in PCa is unclear.

This study used GEO data, CRISPR screens, RNA-seq, single-cell RNA sequencing (scRNA-seq), and in vivo experiments to explore RPS6KC1's role on Enz resistance in PCa. Our study identified *RPS6KC1* as one of key genes in Enz resistance. We found that Warburg effect induces H3K18 lactylation, which regulates the expression of *RPS6KC1* via the transcription factor NF-κB p65. Elevated expression of RPS6KC1 was found to recruit PRDX3 to the mitochondria, thereby mitigating ferroptosis. These findings suggest that the H3K18la/NF-κB/RPS6KC1/PRDX3 axis is important for the development of resistance to Enz. Our findings suggest that the combination of Enz with targeting RPS6KC1 or the ferroptosis inducer Erastin may represent a promising therapeutic strategy for PCa patients exhibiting resistance to Enz.

## Materials and methods

### Cell lines and compounds

LNCaP cells (RRID: SCSP-5021) were obtained from the Cell Bank of the Chinese Academy of Sciences, Shanghai. Enzalutamide (S1250), BAY 11–7082 (S2913), and Erastin (S7242) were purchased from Selleck Chemicals, Shanghai. Additional details are in the supplement materials.

### GEO expression profile analysis

The data were sourced from the GEO database, and the platform's annotation information was employed to convert probes into gene symbols. The GSE221603 dataset by Byula Jee et al. [17], comprising 9 scRNA-seq samples from 7 patients-including 2 metastatic hormone-sensitive prostate cancer (mHSPC) samples and 7 PCa samples-was utilized to investigate the heterogeneity of tumor cells. The GSE278936 dataset by Kiviaho A et al. [18], comprising 48 scRNA-seq samples including 4 benign prostatic hyperplasia sample (BPH), 17 treatment-naïve prostate cancer sample (TRNA), 22 neoadjuvant-treated prostate cancer sample (NEADT), and 5 castration-resistant prostate cancer sample (CRPC)-was utilized to validate the metabolic alternation in tumor cells. Subsequently, gene expression profiles from GSE104935 [19], GSE148397 [20], and GSE70770 [21] were downloaded to examine the expression of hub genes. The acquisition and utilization of the data adhered to the principles and guidelines established by the GEO databases. Additional details are in the supplement materials.

### Data analysis of scRNA-seq

scRNA-seq data were acquired from the GSE221603 and GSE278936 datasets. The study encompassed 21,836 cells from GSE221603 and 120,741 cells from GSE278936, with gene detection per cell ranging from 0 to 20,000. Unsupervised cell clustering was conducted utilizing Principal Component Analysis (PCA) and Uniform Manifold Approximation and Projection (UMAP). Additional details are in the supplement materials.

### In vivo mice model xenograft experiments

Animal procedures were approved by the Animal Ethics Committee at Shanghai Sixth People's Hospital. Male BALB/c nude mice were obtained from Super-B&K Laboratory Animal Corp. Ltd., Shanghai, China, and kept in SPF barrier facilities. Briefly, in the study examining the effects of Enz combined with Erastin treatment, four-week-old BALB/c male nude mice were subcutaneously inoculated with 1 × 10^7^ LNCaP-EnzR cells (shNC or sh*RPS6KC1*). The Enz treatment was administered at a dosage of 20 mg/kg thrice weekly. The Erastin treatment was administered at a dosage of 20 mg/kg/day via intraperitoneal injection. Further details are in the supplement materials.

### ChIP assay, Western blotting, qPCR, IHC and IF assay

Additional details are in the supplement materials.

## Statistics

Statistical analyses were performed with GraphPad Prism 9.0, using Student’s *t*-test or ANOVA for group comparisons. Results are shown as means ± SD, with significance at *p* < 0.05. More details are in the supplementary materials.

## Results

### Integrated CRISPR screen identify *RPS6KC1* as a novel essential gene in Enz resistance

**Fig. 1:**
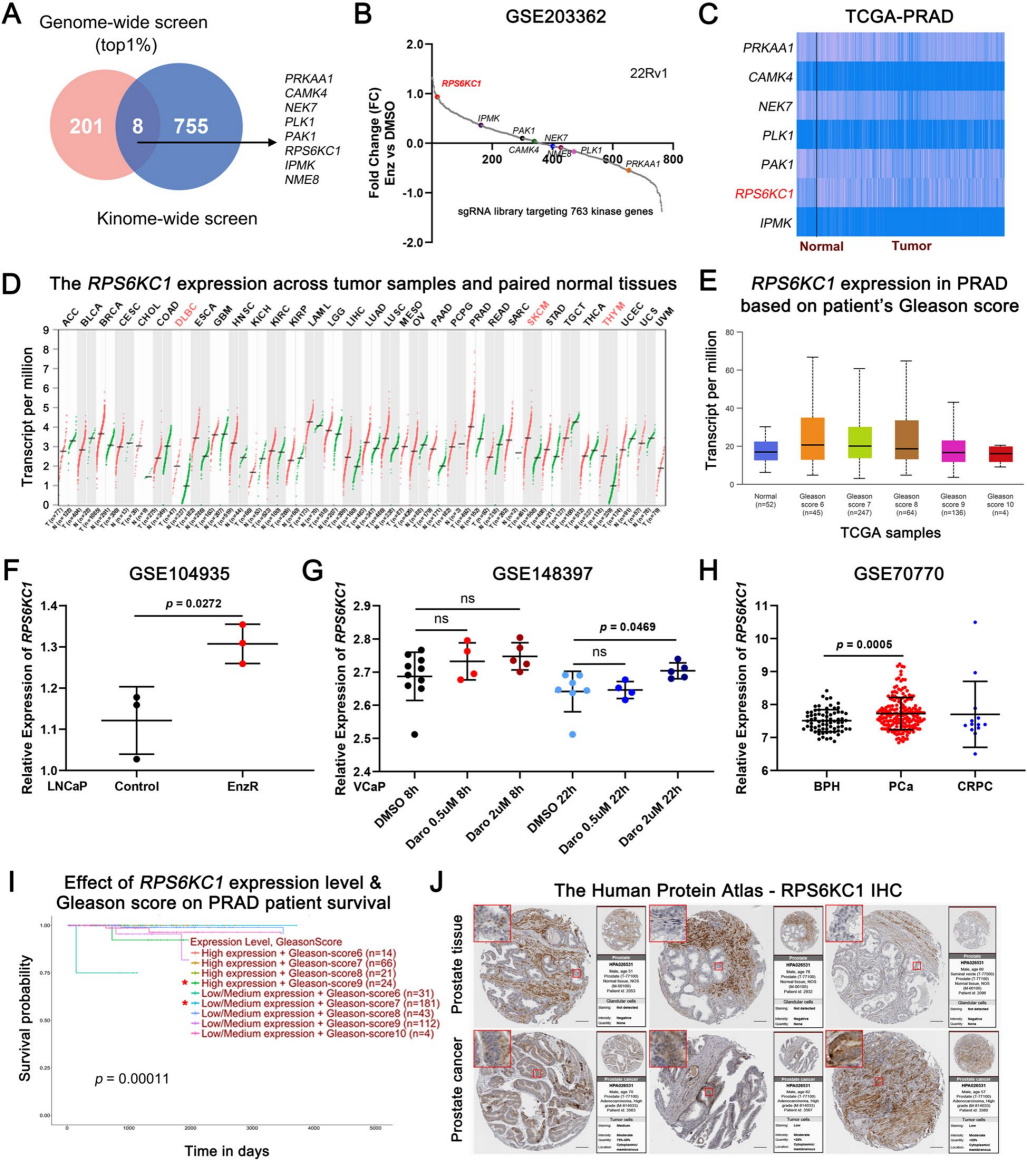
Integrated CRISPR screen identify *RPS6KC1* as a novel essential gene in Enz resistance. **A** The Venn diagram illustrates the integrated analysis of CRISPR genome-wide and kinome-wide screens. The results from the CRISPR kinome-wide screen, which includes 763 human kinases from dataset GSE203362, were analyzed by identifying overlaps with the top 1% of genes ranked in the CRISPR genome-wide screen. Eight genes were found to overlap between the two CRISPR datasets. **B** These overlapping genes were plotted to validate our findings, tracing back to the source dataset GSE203362, with the candidate gene being highlighted. **C** The expression levels of the overlapping genes were examined within the TCGA-PRAD dataset. **D** The expression of *RPS6KC1* was analyzed across multiple tumor types in the TCGA database. **E** The transcriptional levels of *RPS6KC1* across various Gleason scores in the TCGA-PRAD dataset. **F**–**H** The relative gene expression of *RPS6KC1* is compared between control and EnzR groups in the GSE104935 dataset (**F**), between DMSO and Darolutamide (Daro) treatments in the GSE148397 dataset (**G**), and among BPH, primary tumor, and CRPC in the GSE70770 dataset (**H**). **I** A Kaplan–Meier plot illustrating the impact of *RPS6KC1* expression levels and Gleason scores on the survival of patients with PRAD. **J** IHC images of RPS6KC1 in PCa and normal prostate tissues sourced from the HPA database. Statistical analyses included a two-tailed Student's *t*-test (**F**, **G**, and **H**), and a log-rank test (**I**). Error bars represent ± SD

To systematically identify Enz resistance genes in PCa, a comparative analysis was conducted utilizing both CRISPR genome-wide screen and kinome-wide screen to pinpoint common essential genes implicated in Enz resistance. As previously described [22], a pooled CRISPR/Cas9 genome-wide knockout screen was performed in LNCaP EnzR cells. The RIGER statistical algorithms were employed to prioritize screening hits through RNAi Gene Enrichment Ranking. The top 1% of genes, comprising 209 genes based on the RIGER score, were identified as negatively selected genes potentially associated with Enz resistance. Subsequently, we incorporated a sgRNA library from GSE203362, comprising 3,052 unique sgRNAs targeting 763 human kinases, to conduct a comprehensive kinome-wide analysis aimed at identifying kinases whose inhibition could potentially counteract Enz resistance in PCa treatment [23]. Among these, eight kinase genes, *PRKAA1*, *CAMK4*, *NEK7*, *PLK1*, *PAK1*, *RPS6KC1*, *IPMK*, and *NME8* were found to overlap between the two CRISPR datasets (Fig. 1A). Consistent with prior research, these eight essential genes have been reported to have significant associations with the progression of PCa and resistance to Enz [24–26]. To further substantiate our findings linked to the source GSE203362, we employed statistical algorithms via MAGeCKFlute to prioritize screening hits [27]. Among the kinase genes identified, *RPS6KC1* emerged as the top candidate among eight genes (Fig. 1B).

To substantiate the significance of the eight kinase genes, the gene expression analysis was expanded to include the TCGA clinical PCa dataset. The analysis revealed that *PRKAA1*, *NEK7*, *PAK1*, and *RPS6KC1* exhibited elevated expression levels in PCa samples, whereas *NME8* demonstrated such low expression that it was not represented in the heatmap (Fig. 1C). Furthermore, *RPS6KC1* was found to be highly expressed across multiple tumor types, including prostate adenocarcinoma (PRAD) and skin cutaneous melanoma (SKCM) (Fig. 1D). Considering the focal distribution characteristic in PCa tumorigenesis and the critical role of the Gleason scores in risk stratification, we conducted an in-depth analysis of *RPS6KC1* expression levels across different Gleason grades. Our analysis, utilizing a pooled cohort from The Cancer Genome Atlas (TCGA), revealed that *RPS6KC1* is significantly overexpressed in PCa patients compared to those with benign prostatic hyperplasia (BPH). Furthermore, elevated *RPS6KC1* expression was detected in primary PCa patients across a range of Gleason scores (6, 7, 8, 9, and 10) (Fig. 1E).

Considering the potential link between ARSI resistance and PCa progression, we hypothesized shared genomic changes driving both. To explore the mechanisms of PCa resistance to ARSIs, we analyzed *RPS6KC1* expression in cells treated with Enzalutamide (Enz) or Darolutamide (Daro) using two transcriptomic datasets (GSE104935 and GSE148397) and a clinical PCa dataset (GSE70770). The findings indicated that *RPS6KC1* expression was significantly elevated in ARSI-treated cells compared to vehicle-treated cells within the LNCaP and VCaP cell lines, as observed in datasets GSE104935 and GSE148397, respectively. Additionally, this expression pattern was noted in PCa tissue samples in dataset GSE70770 (Fig. 1F-H). In alignment with these results, our analysis confirmed that patients exhibiting high *RPS6KC1* levels coupled with a Gleason score of 9, or those with low to medium *RPS6KC1* levels and a Gleason score of 7, were associated with poor prognosis in PCa samples, according to TCGA data. This suggests the presence of other unexplored mechanisms linking *RPS6KC1* expression with PCa progression that warrant further investigation (Fig. 1I). To thoroughly test our hypothesis, we analyzed protein expression on BPH and PCa patient samples using data from The Human Protein Atlas (HPA). Our results showed higher RPS6KC1 expression in PCa tissues compared to BPH tissues using HPA data (Fig. 1J).

### PCa tissue characteristics analysis by scRNA-seq

Enz resistance is frequently associated with tumor metastasis and represents the primary cause of mortality in PCa, thereby significantly reducing surgical options for patients with advanced stages of the disease. Since AR is crucial in hormone-sensitive PCa, studying AR-reactive tumor cell subclusters in PCa samples using scRNA-seq data could enhance our understanding of Enz resistance. Thus, we aimed to thoroughly profile the molecular landscape of metastatic PCa and non-metastatic PCa to identify biomarkers and therapeutic targets to combat Enz resistance. The GSE221603 dataset, containing 9 PCa tissue samples from 7 patients, was used for scRNA-seq analysis. After processing the data, 2,000 highly variable genes were chosen for further study. Our goal is to analyze tumor cell subclusters with consistent Enz responses across different patients and identify potential combination therapy targets to overcome Enz resistance.

**Fig. 2:**
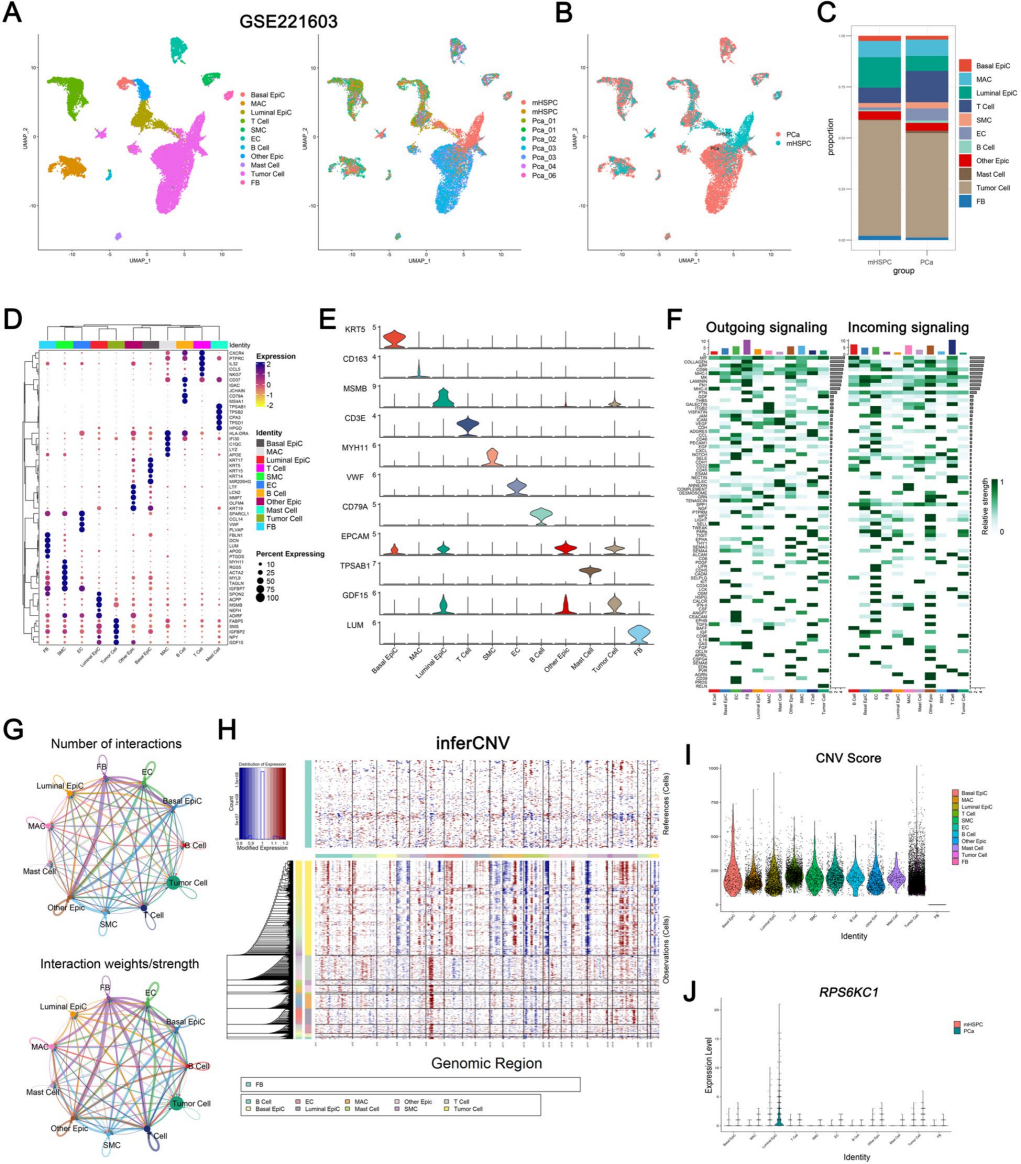
PCa tissue characteristics analysis by scRNA-seq. **A** UMAP visualization reveals cells divided into eleven distinct clusters, each identified by a unique color corresponding to its phenotype. **B** A graph represents the distribution of these eleven cell clusters across two sample groups. **C** A graph illustrates the distribution of cell proportions across different samples, with the vertical axis representing the proportion of each cell type. **D** A heatmap displays the top five DEGs within each cell cluster. **E** Stacked violin plots highlight the most significant variable genes within each cluster. **F** The heatmap illustrates the CellChat signaling dynamics across each cluster. The left panel depicts the outgoing signaling patterns, represented by the expression weight values of signaling molecules, while the right panel illustrates the incoming signaling patterns, indicated by the expression weight values of signaling receptors. The gradient from white to dark green signifies a range from low to high expression weight values within the heatmap. **G** The diagram presents the number of interactions among eleven distinct cell clusters. The line width corresponds to the number of interaction pairs, and varying colors denote different signaling sources. **H** Chromosomal landscape of inferred CNVs among eleven distinct types. **I** Feature plots illustrate the inferred CNV scores across eleven distinct types. **J** Violin plots showing the expression level of *RPS6KC1* gene among the eleven cell clusters across two sample groups

We analyzed 21,836 cells, each with a median of 25,926 expressed genes. Using"RunPCA"and"UMAP"for dimensionality reduction, we identified eleven clusters at a 0.5 resolution. The"SingleR"function classified these into eleven distinct subclusters: Tumor cell, Basal Epicell, Luminal Epicell, Other Epicell, MAC, T cell, SMC, EC, B cell, Mast cell, and FB, distinguished by unique marker genes (Fig. 2A). We then divided these samples into'PCa'and'mHSPC'groups to investigate the characteristics of cell clusters (Fig. 2B). The analysis identified significant differences in the distribution of cell clusters between PCa patients with and without tumor metastasis. Specifically, T cells, endothelial cells (ECs), smooth muscle cells (SMCs), and mast cells were markedly less abundant in the mHSPC group compared to the PCa group, and the Luminal Epicells and Tumor cells were more abundant in the mHSPC group compared to the PCa group (Fig. 2C). These findings highlight the significant impact of metastasis status on the cellular makeup of tumor tissues in PCa patients. The heatmap and stacked violin plots identified key variable genes in each cluster (Fig. 2D-E).

Additionally, a detailed analysis of CellChat's visualization outputs showed that FB, SMC, Luminal Epicell, and Tumor cells mainly send signals, whereas T cells, B cells, MAC, and Mast cells primarily receive signals. In contrast, EC, Basal Epicell, and Other Epicell act as both signal senders and receivers. Bidirectional markers like CDH5, NOTCH, CD34, and JAM are shown (Fig. 2F). The circle plot uses different colors to represent cell interactions. An analysis of interactions among the eleven clusters was conducted, exploring their roles as targets and sources using similar methods (Fig. 2G). Subsequently, although the harmonious integration facilitated a notable separation and fusion of diverse samples, no significant differences were detected in the distribution of tumor cells. As a result, we performed an InferCNV analysis to elucidate the copy number variation (CNV) scores across different cell types based on FB (Fig. 2H). The InferCNV analysis indicated that tumor cells exhibited the highest CNV scores among the cell types examined (Fig. 2I). Following this, we investigated the expression of the identified gene *RPS6KC1* across various cell clusters. The PCa group showed higher average *RPS6KC1* expression levels than the mHSPC group in all cell clusters. Notably, the Luminal Epicell cluster exhibited the highest *RPS6KC1* levels, suggesting a potential link between RPS6KC1 and AR, as Luminal cells are AR-dependent (Fig. 2J).

### Highly malignant tumor cells identification

**Fig. 3:**
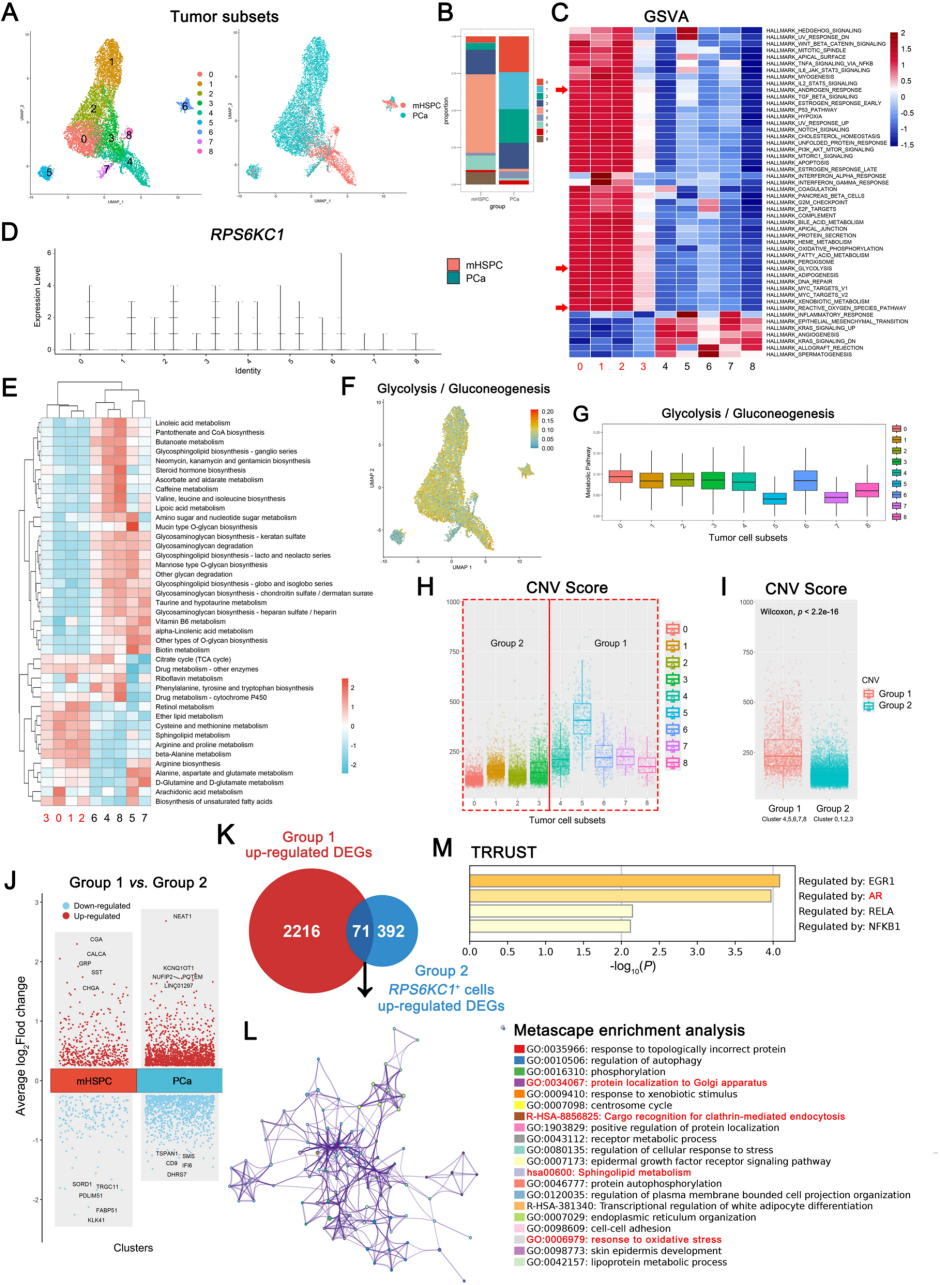
Highly malignant tumor cells identification. **A** The UMAP plot illustrates the PCA clustering outcomes of tumor cell subclusters and sample clustering. **B** A graph depicts the distribution of tumor cell proportions across distinct samples, with the vertical axis representing the proportion of each cell type. **C** A heatmap presents pathways enriched in the nine tumor cell clusters using GSVA, with columns representing cell groups and rows indicating pathways. **D** Violin plots display the expression levels of the *RPS6KC1* gene across the nine tumor cell clusters. **E** The heatmap provides a comparison of multiple metabolism pathway scores among the nine tumor cell clusters. **F** The UMAP visualization depicts the scores of the Glycolysis/Gluconeogenesis pathway. **G** The scores of the Glycolysis/Gluconeogenesis pathway are compared across nine distinct tumor cell clusters. **H** Feature plots display the inferred CNV scores within the nine tumor cell clusters. **I** A comparative analysis of infer CNV scores is conducted between CNV Group1 and Group2. **J** A volcano plot illustrates the differential gene expression between Group1 and Group2 tumor cells in mHSPC and PCa samples. **K** A Venn diagram visualizes the overlap of 71 common hub genes shared between the up-regulated DEGs in Group1 and the up-regulated DEGs in *RPS6KC1*^+^ cells of in Group2. **L** The functional enrichment analysis of 71 common hub genes. **M** The TRRUST enrichment analysis from 71 common hub genes. The horizontal axis represented the *p*-value of GO terms on Metascape

To further characterize the subclusters of PCa tumor cells, we utilized UMAP for dimensionality reduction to investigate the heterogeneity of tumor cells between the PCa and mHSPC groups. The UMAP analysis identified nine transcriptionally distinct tumor cell subclusters (Fig. 3A). Subsequently, we conducted a comparative analysis of the proportions of these tumor cell subclusters. Notably, the relative abundances of subclusters 4, 6, and 8 were significantly elevated in the mHSPC group compared to the PCa group. Conversely, the relative abundance of tumor cell sub-populations 0, 1, 2, 5, and 7 was markedly reduced in the mHSPC group compared to the PCa group (Fig. 3B). To elucidate the principal signaling pathways within each cluster, we conducted Gene Set Variation Analysis (GSVA) across clusters. The analysis revealed an up-regulation in clusters 0, 1, and, 2 of the HALLMARK_ANDROGEN_RESPONSE, HALLMARK_GLYCOLYSIS, and HALLMARK_REACTIVE_OXYGEN_SPECIES_PATHWAY (Fig. 3C). Following this, we investigated the expression of the *RPS6KC1* across nine tumor cell clusters (Fig. 3D). Despite the relatively small number of *RPS6KC1*⁺ cells, which resulted in a scattered dot distribution insufficient to support a violin-like pattern, significant differences in *RPS6KC1* expression were observed between the nine tumor cell clusters in the PCa group and the mHSPC group. Besides, to deepen the understanding of the correlation between metabolic pathways and cellular malignancy, this study identified several metabolic pathways linked to distinct tumor cell subclusters. An analysis of metabolic activity across these subclusters revealed that nearly all exhibited significant metabolic activity in most of the identified pathways (Fig. 3E). Importantly, nearly all tumor cell subclusters showed heightened activity in the tricarboxylic acid (TCA) cycle and drug metabolism via cytochrome P450. Remarkably, a notable proportion (16 out of 41) of pathways exhibited divergent expression patterns in clusters 0, 1, 2, and 3 compared to other clusters, indicating a distinct metabolic alteration among tumor clusters. Furthermore, considering the pivotal role of the Warburg effect in the progression of solid tumors [28], our analysis focused on the glycolysis-related pathway. The results demonstrated that the Glycolysis/Gluconeogenesis pathway showed increased activity across nearly all tumor cell subclusters (Fig. 3F-G).

Previous research shows a positive link between CNV levels and PCa malignancy [29]. InferCNV analysis revealed that tumor cell clusters 4–8 had higher CNV scores, while clusters 0–3 had lower scores (Fig. 3H). We then categorized the tumor cell subclusters into Group 1 and Group 2 based on these scores (Fig. 3I). Group 1 encompasses clusters 4, 5, 6, 7, and 8, while Group 2 includes clusters 0, 1, 2, and 3. To identify genes associated with malignancy in the progression of prostate cancer, differential expression gene (DEG) analyses were conducted between the tumor cell clusters of Group 1 and Group 2 within pooled cells of the mHSPC group and the PCa group (Fig. 3J). We identified a total of 2287 up-regulated DEGs from Group 1, which are highly expressed in high-CNV score malignant tumor cells compared to low-CNV score cells, and may be involved in the progression of advanced prostate cancer.

Next, given that the expression of the *RPS6KC1* is higher in the PCa group compared to the mHSPC group among all cell clusters within Group 2, we initially considered these highly expressed genes in *RPS6KC1*⁺ cells from Group 2 as potential upstream or downstream targets that might be regulated by or associated with RPS6KC1. Despite the overall CNV scores of Group 2 being lower than that of Group 1, the expression of *RPS6KC1* in Group 2 is consistently higher in the PCa group than in the mHSPC group across all cell clusters. This makes Group 2 a more suitable candidate for investigating the function of the *RPS6KC1* gene and enhances our understanding of its role in non-metastatic PCa samples. Therefore, we compared *RPS6KC1*⁺ cells and *RPS6KC1*⁻ cells from Group 2 and identified 463 up-regulated DEGs.

Subsequently, we intersected the 2287 up-regulated DEGs from Group 1 with those 463 up-regulated DEGs of *RPS6KC1*^+^ cells, resulting in the identification of 71 genes (Fig. 3K). The functional enrichment analysis of the shared genes revealed a significant involvement in processes such as protein localization to the Golgi apparatus, cargo recognition for clathrin-mediated endocytosis, sphingolipid metabolism, and response to oxidative stress (Fig. 3L). These findings corroborate the association between *RPS6KC1* expression and high copy number variation in malignant tumor cells, thereby reinforcing the potential pivotal role of RPS6KC1 in PCa progression. Furthermore, TRRUST analysis indicated a high likelihood that these shared genes are regulated by the transcription factor EGR1, AR, RELA, and NFKB1, which have been reported to be related to PCa progression and Enz resistance [30, 31] (Fig. 3M). These results further evidence the role of RPS6KC1 in cancer progression and the potential involvement of oxidative stress.

### RPS6KC1-related transcriptional patterns identification

Based on previous studies showing that RPS6KC1 interacts with PRDX3 or binds with SPHK-1 in early endosomes and regulates key cellular events, we examined PRDX3 and SPHK-1 expression in mHSPC and PCa samples. The results revealed high *PRDX3* expression in all PCa tumor cell clusters, whereas *SPHK1* expression was much lower in both PCa and mHSPC samples, indicating a high abundance of *PRDX3* in PCa cells (Supplement Fig. 1 A). Another significant discovery revealed that the key gene *RPS6KC1* is co-expressed with *PRDX3* and *SPHK-1*, confirming their interaction (Supplement Fig. 1B). Given that PRDX3 is known as an antioxidant protein involved in inhibiting cellular ferroptosis, we analyzed the correlation between *RPS6KC1* expression and ferroptosis pathway activation in PCa samples using TCGA data (Supplement Fig. 1 C). The heatmap in shows ferroptosis marker gene expression in patients with varying *RPS6KC1* levels, confirming a positive correlation between *RPS6KC1* and ferroptosis pathway activation in PCa patients (Supplement Fig. 1D). However, due to PCa's morphological and molecular diversity, changes in *RPS6KC1* expression might further differentiate PCa cell types and impact Enz resistance progression. We examined the expression of various PCa heterogeneity marker genes across nine clusters, focusing on neuroendocrine, AR signaling, stemness, basal, and luminal markers (Supplement Fig. 1E). Our findings indicated that clusters 0, 1, 2, and 3 predominantly expressed AR signaling and luminal markers in the PCa group compared to the mHSPC group, aligning with the *RPS6KC1* expression pattern. Only cluster 4 in the mHSCP group showed increased neuroendocrine marker expression, aligning with previous findings that metastatic PCa, despite performs AR reactivity, undergoes some neuroendocrine transformation, reducing endocrine therapy effectiveness and advancing disease [32]. Overall, these results suggest that RPS6KC1-related PCa progression primarily affects luminal-like PCa cells, rather than causing basal or neuroendocrine transformation.

**Fig. 4:**
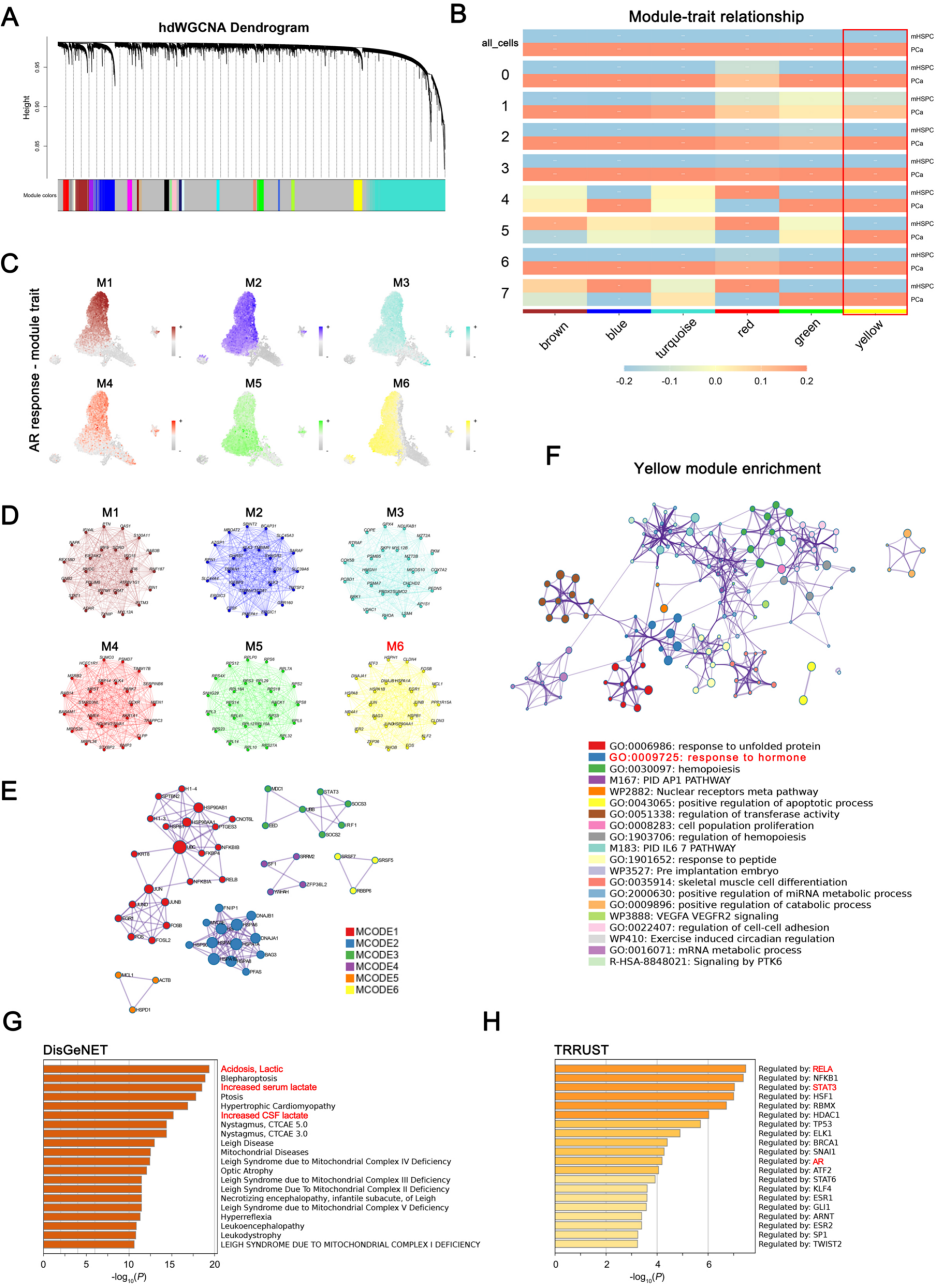
RPS6KC1-related transcriptional patterns identification. **A** Utilizing the optimal soft threshold, a co-expression network was constructed, and genes were organized into distinct modules. The upper section depicted the hierarchical clustering dendrogram, whereas the lower section illustrated the gene modules or network modules. **B** The Plot Module Trait Correlation function within the hdWGCNA package produced a heatmap illustrating the correlation between gene modules and tumor cell clusters. Tumor cell clusters 0, 1, 2, and 3 were consolidated and analyzed as the AR response module-trait. **C** Co-expression network analysis was conducted to compute feature gene-based connectivity, thereby identifying the correlation between cell clusters and the gene module-trait. **D** The first 25 eigengenes core genes of each module and comprehensively compared the connections. **E** MCODE analysis showed the hub gene correlation of each module. **F** The functional enrichment analysis of yellow module hub genes. **G** Enriched DisGeNET terms of yellow module genes. The horizontal axis represented the p-value of GO terms on Metascape. **H** The TRRUST enrichment analysis from the yellow module genes. The horizontal axis represented the p-value of GO terms on Metascape

To investigate RPS6KC1-related transcriptional patterns in PCa, we used the Test Soft Powers function from the hdWGCNA package. By testing various soft thresholds, we determined that a threshold of 6 best maintained a scale-free topology in the co-expression network, accurately representing the biological relationships. In light of the significant role of AR signaling in the progression of PCa and resistance to Enz, we integrated tumor cell sub-clusters 0, 1, 2, and 3, which exhibited AR response, and identified them as key module traits. Subsequently, we examined the interrelationships among gene modules and AR response-module traits within the co-expression network. This analysis assessed gene expression similarity, calculated the topological overlap matrix, and conducted hierarchical clustering, with the co-expression network's hierarchical structure shown in a dendrogram (Fig. 4A). Through the integration of module similarities, we identified six distinct modules associated with eight cell clusters, with the exception of cluster 8, which is exclusive to the mHSPC group, within the mHSPC and PCa groups (Fig. 4B). Notably, the yellow module demonstrates a correlation pattern consistent with the PCa group across each cell subcluster, indicating that the key genes within the yellow module exhibit a stronger correlation with the PCa group. We identified genes with representative expression patterns in each module and calculated their correlation with the module eigenvector, known as module connectivity (kME). The relationships among genes within modules were shown in a dot plot (Fig. 4C). We also extracted and compared core genes from each module, visualizing their relationships in a module-trait co-expression map (Fig. 4D). MCODE analysis also showed the hub gene correlation of each module (Fig. 4E).

Given the strong correlation between the yellow module and AR response, we conducted a gene enrichment analysis of its key genes using Metascape (https://metascape.org/). Key Metascape terms were hormone response, nuclear receptors meta pathway, and transferase activity regulation (Fig. 4F). The analysis performed by DisGeNET demonstrated a significant association between the yellow module hub genes and conditions such as Acidosis, Lactic; Increased serum lactate; and Increased CSF lactate (Fig. 4G), suggesting that these critical hub genes are strongly linked to lactate-related biological processes. The TRRUST analysis suggests that yellow module hub genes are probably regulated by transcription factors RELA, NFKB1, STAT3, and AR, which are linked to lactylation, a novel PTM, and PCa progression (Fig. 4H).

Furthermore, to validate the glycolysis and histone lactylation pathways in other PCa cohorts, we included scRNA-seq data from 48 patient samples, including 4 benign prostatic hyperplasia sample (BPH), 17 treatment-naïve prostate cancer sample (TRNA), 22 neoadjuvant-treated prostate cancer sample (NEADT), and 5 castration-resistant prostate cancer sample (CRPC), from GSE278936. After processing the data, we analyzed 120,741 cells. Using"RunPCA"and"UMAP"for dimensionality reduction, we identified 29 clusters at a 0.5 resolution. The"SingleR"function classified these into nine distinct cell subclusters: Basal Epithelial cell, Luminal Epithelial cell, Other Epithelial cell, Plasma cell, Smooth Muscle cell, Macrophage, Tumor cell, Neutrophil, Fibroblast, distinguished by unique marker genes (Supplement Fig. 2 A). To further characterize the subclusters of PCa tumor cells, we then utilized UMAP for dimensionality reduction to investigate the heterogeneity of tumor cells among TRNA, NEADT, and CRPC groups. We analyzed 14,971 Tumor cells and identified 12 transcriptionally distinct tumor cell clusters (Supplement Fig. 2B). Subsequently, we conducted a comparative analysis of the glycolysis and histone lactylation pathways in these tumor cell clusters (Supplement Fig. 2C-D). Notably, we found relative higher levels of cellular glycolysis and histone lactylation in TC8, TC10, TC11, and TC12, which mainly belong to CRPC and NEADT samples. These results further validate the conclusion that tumor cells may have higher glycolytic activity and histone lactylation in advanced PCa patients.

### RPS6KC1 mitigates ferroptosis and enhances Enz resistance

The main challenge in addressing Enz resistance in PCa patients is identifying genes that cause this resistance, given the significant role of tumor metabolic reprogramming in Enz-related PCa progression [33]. Our earlier study used scRNA-seq to investigate changes in glucose metabolism pathways in Enz-resistant LNCaP EnzR cells, both treated and untreated with Enz [31]. Normally, AR signaling is expected to be inactive under Enz burden. However, according to TRRUST results, TF RELA (P65) might regulate up-regulated DEGs in *RPS6KC1*^+^ cells and yellow module hub genes with AR reactivity. We hypothesized that RELA promotes *RPS6KC1* transcription in Enz resistance development. Thus, we assessed the correlation of expression levels between RPS6KC1 and RELA, RPS6KC1 and PRDX3 utilizing data from TCGA-PRAD. The findings indicated correlation between the expression levels of RPS6KC1, RELA, and PRDX3 (Supplement Fig. 3 A). Then we explored potential binding sites in the *RPS6KC1* promoter by JASPAR. Sequence analysis identified a single P65 candidate (CGGGGTTTCA, R.S. = 0.8011) (Supplement Fig. 3B). ChIP assays showed P65 possessing a binding site in *RPS6KC1* promotor in LNCaP cells, with increased binding in EnzR cells compared to WT cells (Supplement Fig. 3 C).

**Fig. 5:**
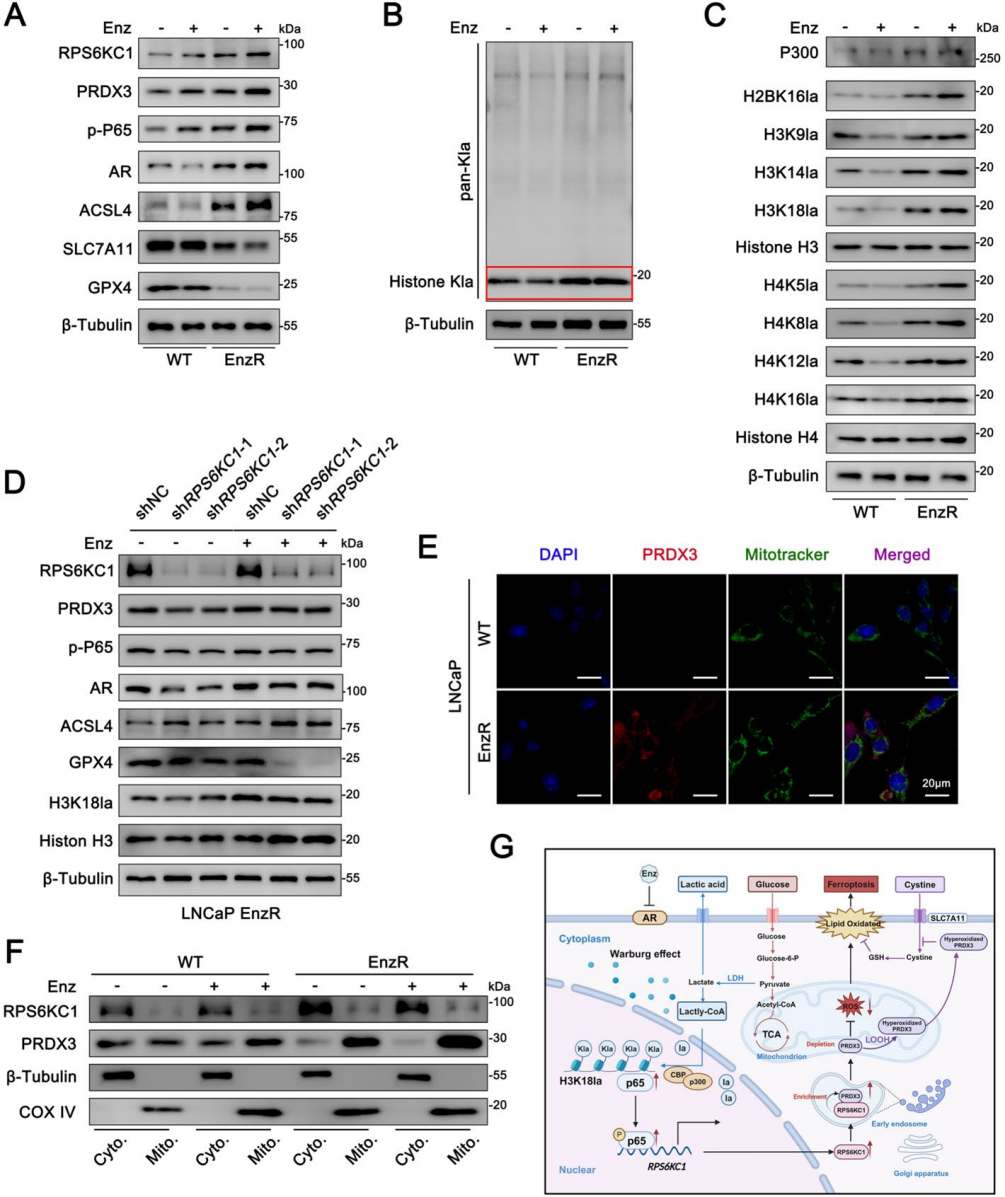
RPS6KC1 alters Enz resistance via H3K18la/P65/RPS6KC1/PRDX3 signaling. **A** The relative levels of RPS6KC protein and key ferroptosis markers in LNCaP WT and EnzR cells, subjected to treatment with or without 10 μM Enz for 72 h, were analyzed using Western blotting. **B** Western blotting was employed to detect Pan-Kla levels in LNCaP cells, with or without Enz burden. **C** Site-specific histone lactylation in LNCaP WT and EnzR cells was analyzed through Western blotting. **D** Western blot analysis was conducted to assess key markers of ferroptosis in LNCaP EnzR cells, both with and without *RPS6KC1* ablation under Enz burden conditions. **E** Representative immunofluorescence images demonstrate the co-localization of Mitotracker and PRDX3 in LNCaP cells. Scale bars represent 20 μm. **F** Assessment of mitochondrial and cytosolic levels of PRS6KC1 and PRDX3 was performed in LNCaP cells under conditions with or without Enz burden. **G** The schematic diagram illustrates the mechanism through which PRS6KC1 mitigates ferroptosis and enhances Enz resistance by recruiting PRDX3

We hypothesize that increased RPS6KC1 may influence PRDX3 expression, affecting drug-resistant cells'response to oxidative stress and leading to Enz resistance. According to prior research indicating that PRS6KC1 interacts with PRDX3 via a pseudo-kinase domain and is subsequently localized to early endosomes [14], in drug-resistant cells, the elevated expression of RPS6KC1 may facilitate the transport of PRDX3, leading to its accumulation in early endosomes and mitochondria. This accumulation potentially mitigates lipid ROS generated by Enz burden, thereby inhibiting ferroptosis. Thus, we conducted Enz burden experiments on LNCaP WT cells and LNCaP EnzR cells. We found the activation of the ferroptosis both in LNCaP WT cells and LNCaP EnzR under Enz burden. Specifically, our results revealed a significant elevation of the key ferroptosis biomarker ACSL4 in response to Enz treatment, suggesting that Enz can induce ferroptosis in prostate cancer. This hypothesis is further corroborated by the marked downregulation of SLC7A11 and GPX4 under Enz exposure. Additionally, our findings indicate that, in the absence of Enz, ACSL4 expression is significantly higher in LNCaP EnzR cells compared to LNCaP WT cells, whereas SLC7A11 and GPX4 expressions are notably lower. These observations imply that the ferroptosis pathway is activated in LNCaP EnzR cells and can be further stimulated by Enz burden (Fig. 5A). This suggests the P65/RPS6KC1/PRDX3 pathway is crucial in reducing ferroptosis and boosting Enz resistance. The enrichment of PRDX3 by RPS6KC1 towards mitochondria may be an important participant in synergistically reducing ROS damage with GPX4.

### RPS6KC1 alters Enz resistance via H3K18la/P65/RPS6KC1/PRDX3 signaling

Recently, the regulation of lactylation, recognized as a novel post-translational modification, has garnered increasing scholarly attention [34–36]. Given that PCa is a type of solid tumor, it is plausible to hypothesize that lactate produced via the Warburg effect may be involved in the regulation of lactylation. Supporting this hypothesis, the single-cell sequencing data from PCa tissue samples presented in this study indicate a potential role for lactylation regulation in the progression of PCa. Indeed, western blot analysis revealed an increase in histone lactylation levels in LNCaP EnzR cells subjected to Enz treatment (Fig. 5B). Subsequently, we quantified lactylation levels at various lysine residues within the histones to pinpoint the specific sites of lactylation in response to Enz. H3K18 lactylation (H3K18la) was markedly increased in EnzR cells subjected to Enz burden compared to WT cells, suggesting a role for H3K18la in the Enz response (Fig. 5C).

Given that prior research has demonstrated that H3K18la in senescent microglia contributes to brain aging and Alzheimer's disease by NF-κB activation [37], we aim to investigate whether RPS6KC1 is implicated in the regulation of H3K18la and its potential impact on the inhibition of cellular ferroptosis and Enz resistance. We used shRNA to reduce *RPS6KC1* expression in LNCaP EnzR cells, resulting in increased ACSL4 and decreased GPX4, indicating ferroptosis activation. H3K18la levels up-regulated with Enz burden (Fig. 5D). Next, to investigate the migration of PRDX3, we assessed the expression and co-localization of Mitotracker and PRDX3 within the LNCaP cells following Enz burden. This was achieved through immunofluorescence (IF) co-localization and mitochondrial/cytoplasmic isolation techniques (Fig. 5E-F). These findings demonstrate that the resistance conferred by RPS6KC1 mitigates ferroptosis and enhances the effects of Enz by recruiting PRDX3 in PCa. Furthermore, consistent with in vitro results, *RPS6KC1* knockdown significantly reduced tumor growth and size under Enz treatment compared to DMSO, especially when combined with Erastin (Supplement Fig. 3D-F). Targeting *RPS6KC1* with a ferroptosis inducer offers a novel approach to overcoming Enz resistance.

## Discussion

Recently, there have been significant advancements in PCa therapy. Enz has been extensively validated for their survival benefits. However, the development of drug resistance continues to pose a major challenge in achieving curative outcomes for PCa patients. Ferroptosis, a regulated cell death pathway induced by iron-catalyzed ROS accumulation, has garnered attention [38]. Emerging evidence indicates that ferroptosis plays a pivotal role in tumor resistance, suggesting that activation of ferroptosis pathways may represent a novel therapeutic strategy to overcome resistance in various cancers. Advanced PCa cells appear to exhibit insensitivity to GPX4 inhibition and ferroptosis induction, contributing to drug resistance and the progression of malignancy. In the present study, we observed that Enz treatment induces lactylation of H3K18, which activates NF-κB p65 and upregulates RPS6KC1 expression. The upregulation of RPS6KC1 facilitates the recruitment of PRDX3 to the mitochondria, where it mitigates ROS-induced damage, reduces ferroptosis, and enhances resistance to Enz (Fig. 5G).

RPS6KC1 features a Phox homology domain, a microtubule-interacting domain, and two pseudo-kinase domains. It is involved in SPP signaling by interacting with SPHK1, an enzyme crucial for sphingosine 1 phosphate production, and directing it to early endosomes. Despite being classified as a kinase, RPS6KC1 functions as a protein transporter rather than exhibiting kinase activity. According to TCGA data (*n* = 496), only patients with high RPS6KC1 levels and a Gleason score of 9 (*n* = 24) or low/moderate RPS6KC1 levels and a Gleason score of 7 (*n* = 181) showed poor prognosis in PCa. This suggests that increased RPS6KC1 expression alone may not drive PCa progression, indicating it may not be the main factor in its carcinogenic role. Since RPS6KC1 lacks kinase function, we focused on the key protein PRDX3 it transports. When EnzR cells were treated with Enz, RPS6KC1 expression significantly increased, while PRDX3 expression showed a slight decrease. Mitochondrial/cytoplasmic separation and IF co-localization revealed that PRDX3 was more expressed in mitochondria, aligning with previous findings of its enrichment in early endosomes by RPS6KC1. PRDX3 then entered mitochondria to help clear ROS, inhibit ferroptosis, and enhance Enz drug resistance.

Our study indicates that histone lactylation, an epigenetic modification, significantly influences Enz resistance and PCa progression. Despite the sample limitations in the GEO datasets, it spans various PCa stages. Future research should explore additional Enz resistance datasets to validate our findings. Moreover, our results suggest alternative pathways to bypass Enz resistance without active AR signaling pathway. Our data indicates that RPS6KC1 expression is positively linked to cellular ferroptosis signaling. Our research suggests that RPS6KC1 inhibits ferroptosis and enhances Enz resistance by recruiting PRDX3 to mitochondria to clear ROS. However, a recent study noted that high oxidative PRDX3 can be a biomarker for ferroptosis in chronic liver disease [16]. Thus, we propose that PRDX3 initially clears ROS upon mitochondrial transport. In ferroptosis process, mitochondrial lipid peroxides cause PRDX3 peroxidation, modifying cysteine thiol groups into sulfinic or sulfonic acids. This prompts PRDX3 to move to the plasma membrane, where it blocks cysteine uptake, causing ferroptosis. Our findings indicate that future studies should investigate PRDX3's role in managing mitochondrial ROS and its effect on Enz resistance.

This study represents the first comprehensive analysis of the role of RPS6KC1 in the progression of PCa through the integration of CRISPR-based genome-wide and kinome-wide screens, scRNA-seq data from tumor tissues, and RNA-seq data from drug-resistant cells. Our findings reveal that the H3K18la/NF-κB/RPS6KC1/PRDX3 signaling axis is crucial in mitigating ferroptosis and contributing to the development of Enz resistance. Furthermore, the ferroptosis inducer Erastin has the potential to counteract this resistance, offering novel insights and therapeutic strategies to address the challenge of Enz resistance in advanced PCa.

## Supplementary Information

Supplementary Material 1.

## Data Availability

No datasets were generated or analysed during the current study.
