# Supplementary material for: Targeting RPS6KC1 to overcome enzalutamide resistance in prostate cancer
Source: Biomark Res. 2025 Aug 23;13:109. doi: 10.1186/s40364-025-00822-x (PMC12375275; doi:10.1186/s40364-025-00822-x)
Supplement: Supplementary file 1 — Supplementary Material 1. [file 40364_2025_822_MOESM1_ESM.docx]

**Targeting RPS6KC1 to overcome enzalutamide resistance in prostate cancer**

**Authors:** Fu-Hao Ji^1,5*^, Yu-Hang Qian^3,4*^, Xiu-Chen Guo^4*^, Hai-Hong Liao^4*^, Jia-Cheng Huang^5*^, Zi-Han Xu^5^, Ming-Ming Yu^6,5^, Yan-Yuan Wu^4#^, Jie-Wen Bao^7#^, Hao-Jie Chen^5,4#^, Yong-Jiang Yu^4#^, Lin Wang^1,2#^

^1^Department of Urology, Ren-Ji Hospital, Shanghai Jiao Tong University School of Medicine, Shanghai, 200001, P.R. China.

^2^Department of Urology, Shanghai Sixth People's Hospital Affiliated to Shanghai Jiao Tong University School of Medicine, Shanghai, 200233, P.R. China.

^3^Department of Urology, Shanghai 411 Hospital, China Rong Tong Medical Healthcare Group Co. Ltd. Shanghai, 200081, P.R. China.

^4^Department of Urology, School of Medicine, Xin-Hua Hospital Affiliated to Shanghai Jiao Tong University School of Medicine, Shanghai, 200092, P.R. China.

^5^Department of Urology, Shanghai Children's Hospital, School of Medicine, Shanghai Jiao Tong University, Shanghai, 200062, P.R. China.

^6^Department of Ultrasound in Medicine, Shanghai Sixth People’s Hospital Affiliated to Shanghai Jiao Tong University School of Medicine, Shanghai, 200233, China.

^7^Department of Urology, Shanghai Ninth People’s Hospital, Shanghai Jiao Tong University School of Medicine, Shanghai, 200011, P.R. China.

**Supplement Material**

**Supplement Figures**

**Supplement Figure 1**

**
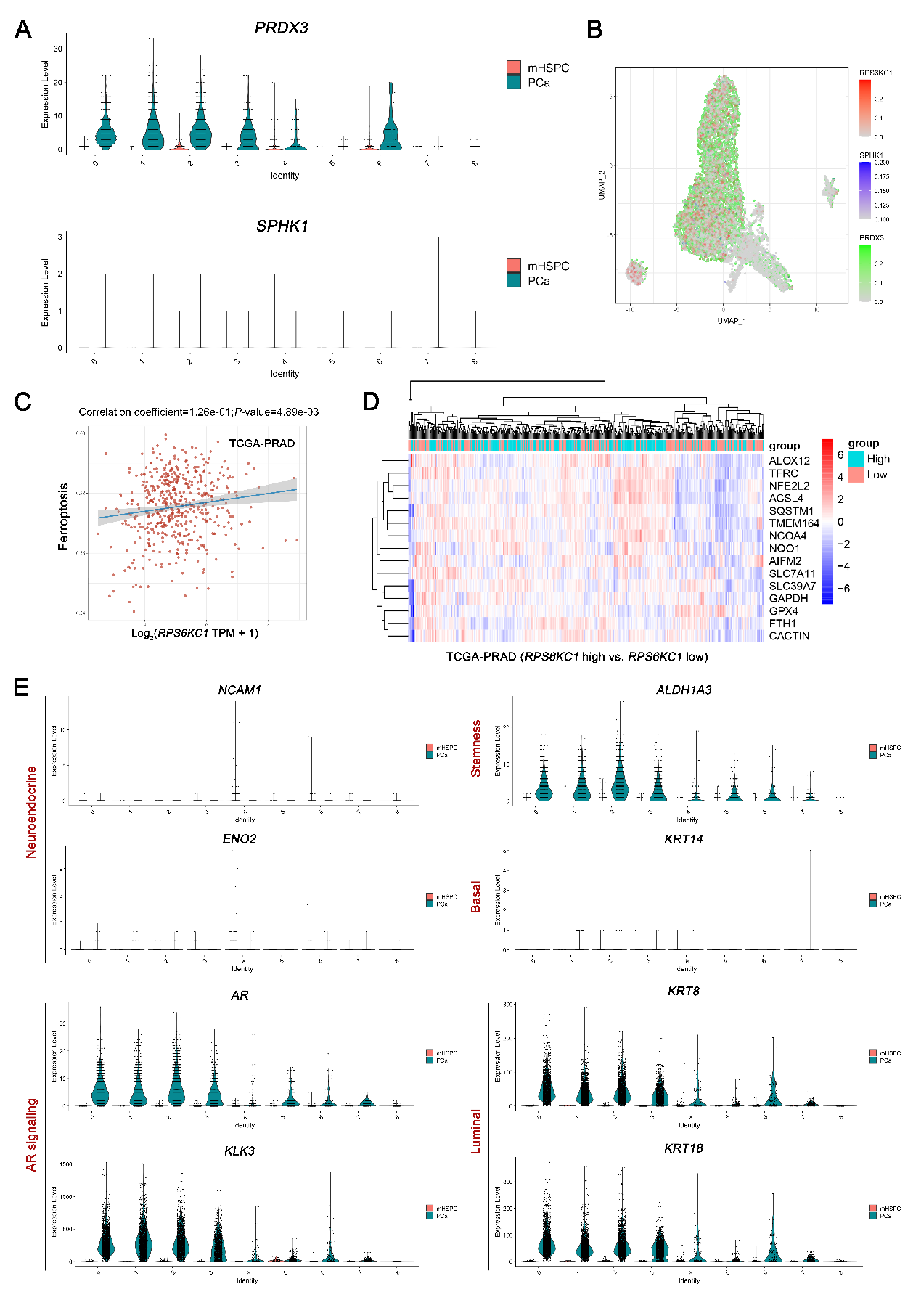
**

**Supplement Figure 1**

(A) Violin plots illustrating the expression levels of PRDX3 and SPHK1 across nine distinct cell clusters. (B) Co-expression feature plots depicting the distribution of RPS6KC1 in co-expression with PRDX3 and SPHK1. (C) Spearman's rank correlation analysis between RPS6KC1 expression and ferroptosis signaling pathways. (D) Heatmaps derived from transcriptomic analysis of 15 ferroptosis marker genes, comparing samples with high versus low RPS6KC1 expression, utilizing data from the TCGA-PRAD dataset. (E) Violin plots showing the expression level of cellular signature genes including neuroendocrine, AR signaling, stemness, basal, and luminal markers among the nine cell clusters across two sample groups.

**Supplement Figure 2**

**
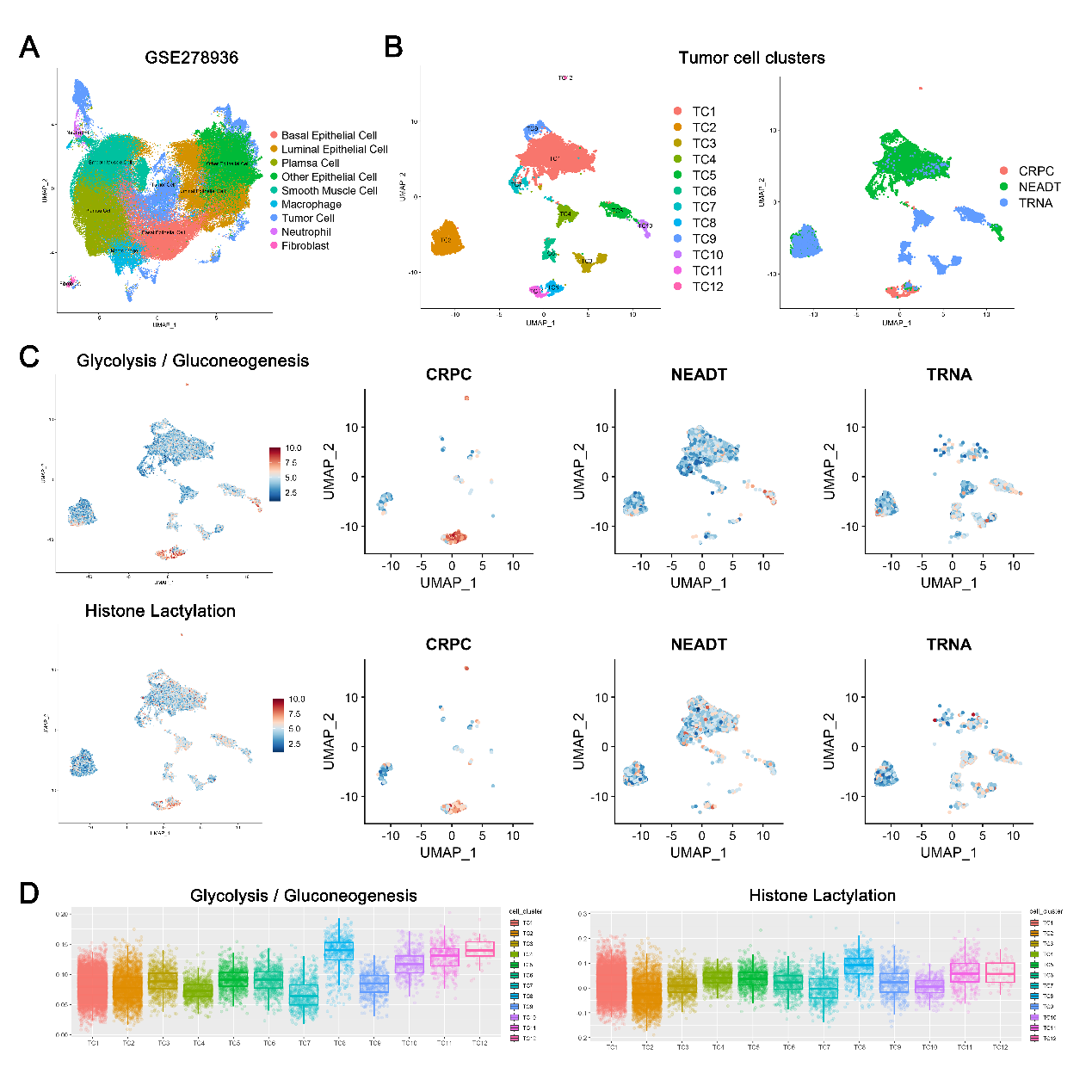
**

**Supplement Figure 2**

(A) UMAP visualization reveals cells divided into 29 distinct clusters, each identified by a unique color corresponding to its phenotype. (B) The UMAP analysis identified 12 transcriptionally distinct tumor cell subpopulations. (C-D) Dot plots (C) and expression comparative analysis (D) of the glycolysis and histone lactylation pathways in tumor cell clusters.

**Supplement Figure 3**

**
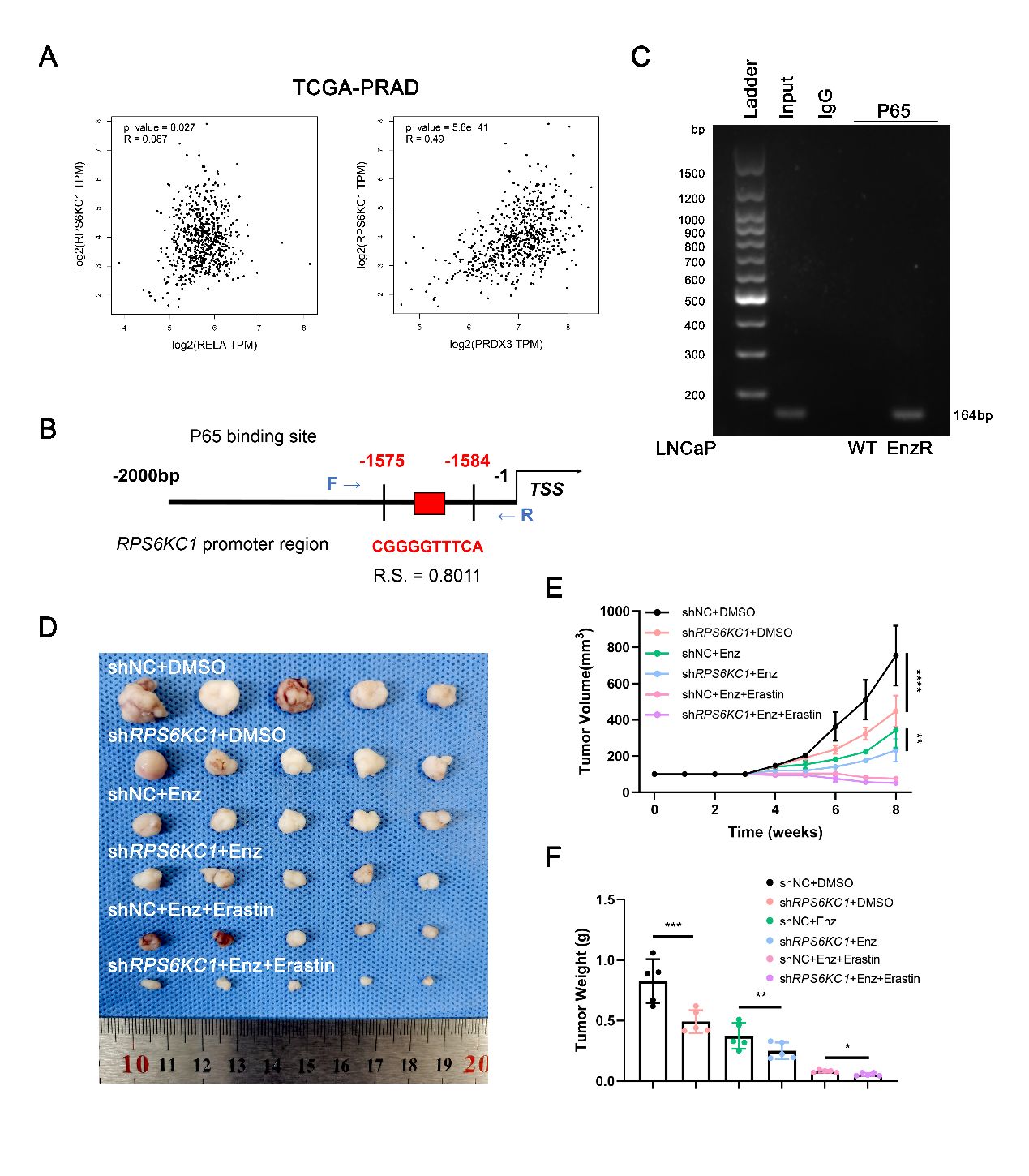
**

**Supplement Figure 3**

(A) Spearman's rank correlation analysis between RPS6KC1 expression and RELA/PRDX3. (B) A potential P65 transcription factor binding site (CGGGGTTTCA) within the promoter region of *RPS6KC1* was identified using the JASPAR database, yielding a relative score of 0.8011 for the candidate motif. (C) Primers F and R were specifically designed to amplify the CGGGGTTTCA motif for ChIP assays. Electrophoretic analysis of ChIP DNA confirmed the binding of P65 to this motif in LNCaP cells. (D) Representative images are provided of tumors xenografted with LNCaP EnzR cells at the conclusion of the study. (E) Tumor growth trajectories of LNCaP EnzR xenografts in castrated mice, with and without Enz treatment, were assessed. Tumor volumes were recorded biweekly and presented as mean ± standard deviation. (F) Post-surgical dissection, tumor weights were measured for each treatment group. Statistical analyses were performed using a two-tailed Student's t-test (J) and one-way ANOVA (E). Error bars represent the ±SD. Statistical significance is denoted as follows: **p* < 0.05, ***p* < 0.01, ****p* < 0.001, *****p* < 0.0001; ns indicates no significant difference.

**Materials and Methods**

**Cell lines and Compounds**

LNCaP cells (RRID: SCSP-5021) were obtained from the Cell Bank of the Chinese Academy of Sciences, located in Shanghai, China. The authenticity of these cells was validated through short tandem repeat (STR) DNA profiling, and they were confirmed to be free from mycoplasma contamination. To reduce potential artifacts associated with the extended culture of immortalized cell lines, cultures were initiated from early passage vials and terminated after a period of 2 to 3 months. Wild-type LNCaP cells were cultured in RPMI 1640 medium (Cytiva, Shanghai, China, SH30809.01). Cells were maintained in culture with the addition of 100 units/mL penicillin (Thermo Fisher Scientific, catalog number 15140122) and 100 units/mL streptomycin (Thermo Fisher Scientific, catalog number 15240062), followed by incubation at 37°C in a humidified chamber with 5% CO_2_. The compounds Enzalutamide (S1250), BAY 11-7082 (S2913), and Erastin (S7242) were procured from Selleck Chemicals, Shanghai. Enzalutamide-resistant LNCaP cells (LNCaP EnzR) were developed by subjecting the parental LNCaP cells to progressively increasing concentrations of enzalutamide (Enz), from 1 µM to 20 µM, over a duration exceeding 12 months, as previously reported.

**GEO expression profile analysis**

Data from the GEO datasets were analyzed, with details in Supplement Table 1. The data were sourced from the GEO database, and the platform's annotation information was employed to convert probes into gene symbols. The GSE221603 dataset, comprising 9 scRNA-seq samples from 7 patients—including 2 metastatic hormone-sensitive prostate cancer (mHSPC) samples and 7 PCa samples—was utilized to investigate the heterogeneity of tumor cells. The GSE278936 dataset comprising 48 scRNA-seq samples including 4 benign prostatic hyperplasia sample (BPH), 17 treatment-naïve prostate cancer sample (TRNA), 22 neoadjuvant-treated prostate cancer sample (NEADT), and 5 castration-resistant prostate cancer sample (CRPC)-was utilized to validate the metabolic alternation in tumor cells. Subsequently, gene expression profiles from GSE104935, GSE148397, and GSE70770 were downloaded to examine the expression of hub genes. The acquisition and utilization of the data adhered to the principles and guidelines established by the GEO databases.

**Data analysis of single-cell sequencing.**

The functionality of candidate genes was validated using single-cell transcriptomic data from dataset GSE221603 and GSE278936. Genes identified in fewer than three cells were excluded from the analysis, and cells with fewer than 200 detected genes were omitted. The number of genes detected per cell ranged from 0 to 20,000, with a total of 21,836 cells from GSE221603 and 120,741 cells from GSE278936 included in the study. Principal component analysis (PCA) was performed utilizing the top 20 principal components as the basis, which subsequently informed the construction of the Uniform Manifold Approximation and Projection (UMAP). Unsupervised cell clustering was conducted utilizing graph-based clustering techniques, which were applied to the top 20 principal components. The Average Expression function was employed to compute the mean expression values for distinct cell subpopulations. The resulting data were visualized using the R package "pheatmap".

**Lentivirus production and plasmid transfection**

To generate cell lines with stable knockdown of *RPS6KC1*, lentiviral vectors incorporating control shRNA and specific sh*RPS6KC1* were designed by Tsingke Biotech Co., Ltd (Shanghai, China). The sequences for the shRNA are provided in Supplement Table 2. To generate lentiviruses, HEK293T cells were transfected with either shRNA or an expression plasmid in conjunction with the GMeasy™ lentivirus Packaging Kit (Gmeasy-40, Genechem Co. Ltd, Shanghai, China). The viral supernatant was collected at 72 hours post-transfection, followed by filtration and concentration of the viral particles through centrifugation.

**Western Blotting Analysis**

Cells were prepared using an immunoprecipitation assay buffer (Thermo Fisher Scientific, 89900), which was supplemented with Halt protease and phosphatase inhibitors (Thermo Fisher Scientific, 78440). Protein concentrations were determined via the bicinchoninic acid protein assay kit (Beyotime Biotechnology, Shanghai, China, P0012). Subsequently, proteins were separated by 10% sodium dodecyl sulfate-polyacrylamide gel electrophoresis and transferred onto polyvinylidene difluoride membranes. A freshly prepared 5% solution of non-fat milk in 1X TBST buffer (composed of 25 mM Tris, pH 7.5, 137 mM NaCl, 2.7 mM KCl, and 1 ml Tween 20) was utilized for blocking for a period of 2 hours at ambient temperature. Subsequently, the membranes were incubated with primary antibodies overnight at 4 °C. Thereafter, the membranes were subjected to three washes with 1X TBST buffer, each with a duration of 5 minutes, followed by incubation with a horseradish peroxidase-conjugated secondary antibody. Membrane visualization was accomplished using enhanced chemiluminescence (ECL) reagents and an ECL Plus detection system. Supplement Table 3 lists all primary antibodies, including their dilutions and vendor details.

**ChIP assay**

LNCaP cells, with a cell count surpassing 1 × 10^7^, were subjected to crosslinking. A 4% formaldehyde solution was introduced into a petri dish containing RPMI 1640 medium, resulting in a final formaldehyde concentration of 1%. The mixture was incubated at 37 °C within a culture chamber for 15 minutes. Following this, a 5M glycine solution was added to the petri dish to achieve a final concentration of 0.125 M, and the cells were further incubated for 15 minutes at room temperature. The cells were subsequently washed twice with phosphate-buffered saline containing 1M PMSF and then collected by scraping into a 1.5 mL Eppendorf tube. The harvested cells underwent centrifugation at 1000g for 5 minutes.

Chromatin immunoprecipitation (ChIP) assays were performed using the ChIP assay kit from Beyotime, following the manufacturer's protocol meticulously. The ChIP procedure utilized an anti-P65 antibody (Abcam, ab32536) or rabbit IgG (Abcam, ab6702) as a negative control, in the presence of 100 µg/mL sonicated salmon sperm DNA, with overnight rotation at 4°C. The reversal of formaldehyde-induced cross-linking was achieved by heating the samples at 65°C overnight. Following this, DNA purification was conducted utilizing the QIAquick PCR Purification Kit (Qiagen, 28106). Input samples were subjected to the same protocol, with the exception of the immunoprecipitation step. A ChIP-PCR assay was subsequently performed. The input control was derived from the supernatants of sonicated lysates. PCR was carried out using primers specific to the P65-binding region within the *RPS6KC1* promoter domain. The sequences of the primers employed are provided in Supplement Table 4.

***In vivo* Mice Model Xenograft Experiments**

All animal procedures were approved by the Shanghai Sixth People's Hospital Animal Ethics Committee, following the Guide for the Care and Use of Laboratory Animals. Four-week-old male BALB/c nude mice were supplied by Super-B&K Laboratory Animal Corp. Ltd and housed in SPF facilities at Shanghai Sixth People's Hospital.

In the study examining the effects of Enz combined with Erastin treatment, four-week-old BALB/c nude mice were subcutaneously inoculated with 1 × 10^7^ LNCaP-EnzR cells (shNC or sh*RPS6KC1*). Following surgical castration, the mice were randomly allocated to various treatment groups (n=5 in each group). The Enz treatment was administered at a dosage of 20 mg/kg thrice weekly. The Erastin treatment was administered at a dosage of 20 mg/kg/day via intraperitoneal injection. Tumor growth was monitored biweekly, and tumor volume was calculated using the formula: 0.52 × length × width^2^. All mice were sacrificed 2 days post-final drug dose, and tumors were then photographed and weighed.

**Statistical analysis**

Statistical analyses and visualizations were performed using GraphPad Prism 9.0. Group comparisons were evaluated using either Student’s *t*-test or ANOVA. The chi-squared (*χ*²) test was utilized to examine associations between groups. Survival curves were constructed using the Kaplan-Meier method. Pearson's correlation analysis was conducted to assess correlations. Data are presented as means ± standard deviations (SD), and a p-value of less than 0.05 was considered indicative of statistical significance.

**Data availability statement**

All the data supporting the findings of this study can be accessed within the article and its Supplement Information files, as well as through a reasonable request to the corresponding author.

**Supplement Table 1. GEO Datasets used**

| Dataset | GSM file | Usage |
| --- | --- | --- |
| GSE203362 | GSM6167542 - GSM6167545 | CRISPR kinome-wide screen |
| GSE104935 | GSM2810318 - GSM2810322 | Hub gene expression validation |
| GSE148397 | GSM4467175 - GSM4467211 | Hub gene expression validation |
| GSE70770 | GSM1817833 - GSM1817976 | Hub gene expression validation |
| GSE221603 | GSM6890190 - GSM6890192,  GSM6890196 - GSM6890204,  GSM6890208 - GSM6890210 | Single-cell RNA-seq profiling for PCa tissue samples |
| GSE278936 | GSM8557976 - GSM8558023 | Single-cell RNA-seq profiling for PCa tissue samples |

**Supplement Table 2. shRNA list**

| shRNA | Sequence (5’-3’) |
| --- | --- |
| sh*RPS6KC1-1* | 5’-CCGGCCCAGCTCAGATCCTAAGTTTCTCGAGAAACTTAGGATCT  GAGCTGGGTTTTTT-3’ |
| sh*RPS6KC1-2* | 5’-CCGGGCAATGAATATGGGCAAGAAACTCGAGTTTCTTGCCCATATT  CATTGCTTTTTT-3’ |

**Supplement Table 3. Antibody list**

| **Antibodies** | **Host** | **Source** | **Identifier** | **Application/Dilutions** |
| --- | --- | --- | --- | --- |
| Anti-P65 | Rabbit | Abcam | #ab32536 | IB 1:1000; 5 µg for 50 µg of chromatin |
| Anti-Rabbit IgG H&L | Goat | Abcam | #ab6702 | 5 µg for 50 µg of chromatin |
| Anti-Phospho-P65 (Ser536) | Rabbit | Abcam | #ab76302 | IB 1:1000 |
| Anti-RPS6KC1 | Rabbit | Abcam | #ab234705 | IB 1:1000; IF 1:100 |
| Anti-PRDX3 | Rabbit | Abcam | #ab128953 | IB 1:1000; IF 1:100 |
| Anti-ACSL4 | Rabbit | Abcam | #ab155282 | IB 1:1000 |
| Anti-AR | Rabbit | Abcam | #ab108341 | IB 1:1000 |
| Anti-SLC7A11 | Rabbit | Abcam | #ab307601 | IB 1:1000 |
| Anti-GPX4 | Rabbit | Abcam | #ab125066 | IB 1:1000 |
| Anti-Pan-Kla | Rabbit | TPM Bio | PTM-1401 | IB 1:1000 |
| Anti-P300 | Mouse | Santa Cruz | sc-32244 | IB 1:1000 |
| Lactyl-Histone Antibody Sampler Kit | Rabbit | TPM Bio | PTM-7093 | H2BK16la, H3K9la, H3K14la, H3K18la, H4K5la, H4K8la, H4K12la, H4K16la, Histone H3, Histone H4; IB 1:1000 |
| Anti-COX IV | Rabbit | Abcam | #ab16056 | IB 1:1000 |
| Anti-β-Tubulin | Rabbit | Abcam | #ab6046 | IB 1:1000 |

**Supplement Table 4. ChIP PCR primer list**

| **ChIP PCR Primer** | **Sequence (5’-3’)** |
| --- | --- |
| *RPS6KC1-F* | TTACAGAAAGGCGCTCAATGG |
| *RPS6KC1-R* | AGTGGAGTGTTGAAGGGATGG |
